# Naptumomab Estafenatox: Targeted Immunotherapy with a Novel Immunotoxin

**DOI:** 10.1007/s11912-013-0370-0

**Published:** 2014-01-21

**Authors:** Tim Eisen, Gunnar Hedlund, Göran Forsberg, Robert Hawkins

**Affiliations:** 1 Cambridge University Health Partners, Addenbrooke’s Hospital, Cambridge, UK; 2 Active Biotech AB, Lund, Sweden; 3 The Christie NHS Foundation Trust, Manchester, UK; 4 University of Manchester, Manchester, UK

**Keywords:** Immunotherapy, Immunotoxin, Tumor-targeted superantigen, 5T4 tumor antigen, Carcinoma, Kidney cancer, Renal cell carcinoma, Evolving therapies, Naptumomab estafenatox

## Abstract

Improvement of cancer therapy by introducing new concepts is still urgent even though there have been major advancements lately. Immunotherapy is well on the way to becoming an established tool in the cancer treatment armory. It seems that a combination of (1) activation of immune effector cells and selective targeting of them to tumors and (2) the inhibition of immune suppression often induced by the tumor itself are necessary to achieve the therapeutic goal. The immunotoxin naptumomab estafenatox was developed in an effort to activate and target the patient’s own T cells to their tumor, by fusing a superantigen (SAg) variant that activates T lymphocytes to the Fab moiety of a tumor-reactive monoclonal antibody. Naptumomab estafenatox targets the 5T4 tumor antigen, a 72-kDa oncofetal trophoblast protein expressed on many carcinomas, including renal cell carcinoma. The therapeutic effect is associated with activation of SAg-binding T cells. The SAg-binding T lymphocytes expand, differentiate to effector cells, and infiltrate the tumor. The therapeutic efficacy is most likely related to the dual mechanism of tumor cell killing: (1) direct lysis by cytotoxic T lymphocytes of tumor cells expressing the antigen recognized by the antibody moiety of the fusion protein and (2) secretion of cytokines eliminating antigen-negative tumor cell variants. Naptumomab estafenatox has been clinically tested in a range of solid tumors with focus on renal cell carcinoma. This review looks at the clinical experience with the new immunotoxin and its potential.

## Introduction

The therapeutic landscape for many malignancies is rapidly changing. Among the important developments of recent years is the rise of therapies activating or releasing the power of immunological mechanisms. Older immune therapies against solid malignancies have proved toxic and only modestly active in clinical practice. However, a deeper understanding of tumor immunology has led to recent successes, including the FDA approval of sipuleucel-T to treat prostate cancer as well as ipilimumab to treat malignant melanoma. Although certain cancers have been identified as being susceptible to immune-based interventions, new evidence indicates that immunotherapies might become a general therapeutic tool in the treatment of cancer. It seems that a combination of (1) activation of immune effector cells and selective targeting of them to tumors and (2) the inhibition of immune suppression often induced by the tumor itself are necessary to achieve the therapeutic goal. In an effort to activate and target the patient’s own T cells to their tumor, the immunotoxin naptumomab estafenatox was developed and has so far been clinically tested in a range of solid tumors with focus on renal cell carcinoma. This report provides an overview of the clinical experience and the status of development of the naptumomab estafenatox (ABR-217620; ANYARA).

## Naptumomab Estafenatox—a Tumor-Targeted Superantigen

The tumor-targeted superantigen (TTS) concept uses a unique approach to target large numbers of T cells to tumors [1, 2•, 3] and utilizes a distinct type of immunotoxin. T lymphocytes are very effective in eliminating some experimental tumors. However, in humans, tumor-specific T cells often occur in low numbers or may be suppressed. Therefore, their numbers are insufficient to interfere with established growing cancer. In the TTS concept, bacterial superantigens (SAgs), the most potent known activators of human T cells, are used to activate and recruit large numbers of T cells to the targeted tumors.

**Fig. 1:**
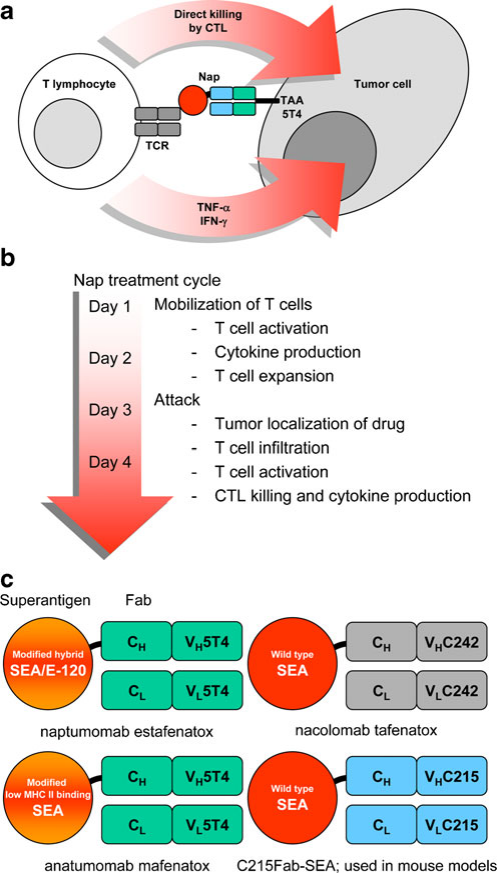
**a** Mechanism of action of naptumomab estafenatox (*Nap*). **b** Immune/drug functions during a naptumomab estafenatox (*Nap*) treatment cycle. **c** Different fusion proteins. The tumor-targeted superantigens are naptumomab estafenatox, anatumomab mafenatox, nacolomab tafenatox, and C215–staphylococcal enterotoxin A (*SEA*). *CTL* cytotoxic T lymphocyte, *IFN* interferon, *TAA* tumor-associated antigen, *TCR* T-cell receptor, *TNF* tumor necrosis factor

The wild-type SAg staphylococcal enterotoxin A (SEA) binds effectively to MHC class II^+^ accessory cells and activates both CD4^+^ and CD8^+^ T cells [4, 5]. MHC class II^+^ cells are killed by cytotoxic T lymphocytes (CTLs) when targeted by SEA [6–9]. A tumor-specific SAg is created by fusing the SAg to the Fab moiety of a tumor-reactive monoclonal antibody (mAb). Fab–SAg fusion proteins have been successfully used to cure mice with established experimental tumors. The therapeutic effect was associated throughout the tumor with a massive infiltration of T cells which actively produced tumoricidal cytokines such as tumor necrosis factor α and interferon (IFN)-γ. The therapeutic efficacy is most likely related to the dual mechanism of tumor cell killing: direct lysis of tumor cells expressing the antigen recognized by the antibody moiety of the fusion protein and secretion of cytokines eliminating antigen-negative tumor cell variants (Fig. 1).

The TTS therapeutic proteins, including naptumomab estafenatox, are typically used in cycles of four to five once-daily intravenous injections. In the first phase of a cycle, the T lymphocytes are activated and differentiate into effector cells, which later in the cycle localize to the tumor and mediate their antitumor functions (Fig. 1). This treatment schedule can be repeated and is easily combined with other anticancer drug modalities.

The early clinical trials with TTS were performed using wild-type SEA as the T-cell activator [10, 11]. However, this fusion protein (nacolomab tafenatox) was associated with a most potent T-cell activation and therefore it was necessary to give the intravenous doses at only a few nanograms per kilogram to avoid unacceptable toxicity. In addition, the dose had to be individualized on the basis of preformed anti-SAg (anti-SEA) antibodies. Wild-type SAgs bind to MHC class II cell membrane proteins, and the complexes subsequently bind to T-cell receptors (TCRs) containing certain Vβ sequences. After proliferation and differentiation, these T lymphocytes infiltrate the tumor tissue and are activated in the tumor owing to TCR binding to the SAgs of the TTS protein. The activated T cells produce cytokines, mainly of the T_h_1 type, e.g., IL-2 and IFN-γ, and perform direct perforin-dependent tumor killing. To achieve maximal targeted antitumor effects, a balanced relationship between the binding affinities of the three functional binding sites in the TTS is required. The desire to mimic the T-effector cell (e.g., the CTL) binding via its TCR to the MHC/tumor peptide target on a tumor target cell drove the development of naptumomab estafenatox, which showed distinct characteristics regarding binding to the tumor antigen (5T4), the TCR, and the MHC class II proteins [12••, 13]. It is currently hypothesized that an optimal TTS should have very high affinity for the tumor antigen, low affinity for TCR, and very low affinity for MHC class II proteins. Naptumomab estafenatox binds to the 5T4 tumor antigen with *K*_D_ of approximately 1 nM. SEA/E-120, the SAg part of naptumomab estafenatox, has been characterized in detail and has been found to primarily bind and operate through TCR TRBV7-9 with a binding constant of 0.48 μM and an affinity for MHC class II proteins of 51 μM.

A fusion protein consisting of the Fab moiety of the mAb 5T4 fused to SEA with a point mutation decreasing MHC class II protein binding (associated with decreased toxicity), anatumomab mafenatox [14], a predecessor of naptumomab estafenatox, was investigated in clinical phase 1 and phase 2 trials with encouraging results [15, 16•]. However, this fusion protein was also associated with some of the drawbacks seen with nacolomab tafenatox, particularly the need to give low doses as well as individualized dosing based on preformed anti-SAg (anti-SEA) antibodies. The novel genetically modified immunotoxin naptumomab estafenatox displays strong affinity for the 5T4 tumor antigen, on the order of 1 nM, and is approximately 50 % more efficient than anatumomab mafenatox in killing human 5T4-positive tumor cells in vitro [12••]. Naptumomab estafenatox does induce an MHC class II and dose dependent proliferation of human lymphocytes, but only at 50-fold and more than 10^3^-fold higher concentrations than anatumomab mafenatox and nacolomab tafenatox, respectively [12••]. Furthermore, preformed human anti-SAg antibodies did not recognize the functional regions in the SAg part of naptumomab estafenatox to the same extent as in the predecessor construct [12••]. Thus, even though naptumomab estafenatox has very potent antitumor properties, it contains an SAg moiety without classic SAg activity. The MHC class II binding capacity is minimal and the T-cell proliferation induced by naptumomab estafenatox has a median effective concentration more than 10^3^ times higher than those of conventional wild-type SAgs, resulting in dramatically reduced toxicity. Furthermore, the levels of target epitopes for naturally occurring anti-SAg antibodies were reduced and dosing of naptumomab estafenatox was therefore considered to be independent of normal baseline levels of anti-SAg antibodies.

Naptumomab estafenatox targets the 5T4 tumor antigen, a 72-kDa oncofetal trophoblast protein originally isolated from placenta and characterized by the use of the murine IgG1 mAb 5T4 [17, 18]. Expression of 5T4 antigen has been shown to influence adhesion, cytoskeletal organization, and motility, properties which might account for its association with poorer clinical outcome in some cancers [19]. A general screen by immunohistochemistry on frozen sections showed that many different carcinomas express the 5T4 antigen [20–27] (Table 1), and very often the 5T4 antigen levels appear to be higher in more advanced stages of cancer. Studies have shown a homogenous reactivity, which in most cases is moderate to strong, in more than 95 % of non-small-cell lung cancer (NSCLC), pancreatic cancer (PC), and renal cell cancer (RCC). In many cases, tumor stromal reactivity has been observed in addition to tumor cell reactivity. Outside the placenta, 5T4 normal tissue reactivity is considered to be most limited and there is no evidence of circulating 5T4 antigen. Naptumomab estafenatox with the selectivity of the 5T4 mAb has been in clinical development primarily for the indications of NSCLC and RCC. It targets a tumor antigen highly expressed on tumor cells, with very low expression on normal tissue and without soluble antigen interference.

**Table 1:** 5T4 reactivity in a variety of tumor types *Cancer*

*Cancer*	***Stage***/***Type***	***Reactivity***	***Conclusions***
Breast	Mixed	56/63	85–95 % of patients express the 5T4 antigen.
Cervix	Mixed	5/5	85–90 % of patients express the 5T4 antigen.
	I	22/25	
	II	22/26	
	III	9/10	
	IV	5/6	
Colorectal	Mixed	11/22	40–50 % of patients express the 5T4 antigen. In Dukes stage D patients, more than 70 % over-express the antigen.
	A	2/8	
	B	7/34	
	C	13/21	
	D	7/9	
Gastric	Mixed	41/93	Approximately 50 % of patients express the 5T4 antigen on tumor cells. Most samples with 5T4 negative tumor cells have 5T4 positive tumor stroma [25].
	I	1/2	
	II	1/4	
	III	1/1	
	IV	12/20	
NSCLC	Mixed	259/261	99 % of patients express the 5T4 antigen.
Ovarian	Mixed	4/7	More than 70 % of patients express the 5T4 antigen.. For stage IV patients, 90–95 % of the patients are 5T4 positive.
	I	2/10	
	II	4/57	
	III	21/29	
	IV	24/26	
Pancreatic	Mixed	23/23	100 % of patients express the 5T4 antigen.
Prostate	Mixed	26/26	100 % of patients express the 5T4 antigen.
Renal	Clear cell	215/222	>95 % of patients express the 5T4 antigen.
	Papillary	18/18	
	Mixed	36/37	

## Naptumomab Estafenatox—Phase 1 Studies

Naptumomab estafenatox was developed in parallel with the initial clinical studies with its predecessor anatumomab mafenatox and has been evaluated in phase 1 trials in NSCLC, PC, and RCC patients [28••, 29]. Altogether, 52 patients were investigated in the monotherapy (MONO) trial and the combination therapy (COMBO) trial with docetaxel. The primary aim was to establish the maximum tolerated dose of naptumomab estafenatox. The secondary objectives were to determine the safety profile, pharmacokinetic parameters, immunological response, and effects on tumor disease. The MONO study was conducted in Norway, the UK, and the USA. The main inclusion criteria were histologically or cytologically confirmed disease, which was refractory to available standard therapies. Patients had to have an Eastern Cooperative Oncology Group performance status of 0 or 1 and life expectancy of more that 3 months. Three different indications were investigated, consisting of 20 cases of NSCLC, 11 cases of RCC, and eight cases of PC. The starting dosage was 500 ng/kg/day administrated as a 5-min bolus injection and naptumomab estafenatox was given for five consecutive days. Six dose-limiting toxicities were recorded, and the highest dosage investigated was 27.4 μg/kg/day. Patients with stable disease or response were considered for treatment with a second 5-day cycle, unless they had experienced a dose-limiting toxicity. The most notable side effects were fever, nausea, and rigors, and these were easily managed. It was concluded that the side effects were not dependant on the anti-SAg (anti-SEA/E-120) antibody titers. The recommended phase 2 dosage was set at 15 μg/kg/day for patients with RCC and 22 μg/kg/day for patients with NSCLC and PC.

As part of the immune activation, increased systemic levels of IL-2, IFN-γ, and IL-10 were observed 3 h after naptumomab estafenatox treatment, and there was a correlation between the dose and cytokine induction. We have evaluated the cytokine responses as a surrogate for a beneficial pharmacologic effect [16•] in all patients from the MONO and COMBO studies. Plasma IL-2 levels were dose-dependently increased 3 h after injection of naptumomab estafenatox in the first treatment cycle [28••]. RCC patients had larger increases than NSCLC or PC patients. The IL-2 response declined in the later parts of the treatment cycle, showing only marginally elevated IL-2 levels on days 4 and 5. Plasma IFN-γ levels were also dose-dependently increased 3 h after TTS injection. The IFN-γ response declined only slightly as the treatment cycle progressed, and unlike IL-2, high IFN-γ levels were also recorded on days 4 and 5. Plasma IL-10 levels were dose-dependently increased 3 h after injection of naptumomab estafenatox and continued to increase as the treatment cycle progressed. One may speculate that a powerful immune activation stimulates inhibitory responses, such as IL-10 production, similar to that seen as a vigorous antivirus response is naturally downregulated.

A mechanistically important finding was that the SAg reactive population of T cells expressing the TCR TRBV7-9 was selectively expanded after naptumomab estafenatox treatment and returned to normal levels at day 28. The anti-SAg (anti-SEA/E-120) levels were stable or only moderately increased in approximately 50 % of the patients after one cycle, and human anti-mouse antibody levels were very low.

A major objective in the development of the TTS fusion proteins has been a short systemic exposure to provide high tumor targeting capability combined with acceptable toxicity. The pharmacokinetics of naptumomab estafenatox show a small volume of distribution (about 0.14 L/kg) and a low clearance (about 0.13 L/h/kg). The mean terminal half-life was determined to be 1.4 h. No time dependency was seen in the pharmacokinetics in the first treatment cycle, with similar plasma concentrations on day 1 and day 5. Furthermore, no dose dependency in the pharmacokinetics was indicated at doses of 10 μg/kg and greater and no effect of gender or disease indication in the pharmacokinetic parameters of naptumomab estafenatox was seen. During the first treatment cycle no significant effect of anti-SAg (anti-SEA/E-120) antibody levels on pharmacokinetic parameters was seen .

Efficacy was analyzed by CT scan. Several patients in the MONO study had tumor shrinkage, not amounting to formal Response Evaluation Criteria in Solid Tumors partial responses. The median overall survival times were 15.8 months for the NSCLC patients, 26.2 months for the RCC patients, and 5.2 months for the PC patients. Although these studies were not controlled and a bias in patient selection cannot be excluded, the median overall survival for the NSCLC and RCC patients was encouraging. It should be noted that these early studies were performed prior to the widespread use of antiangiogenic agents in the treatment of RCC.

An open-label dose-escalation study of naptumomab estafenatox in combination with docetaxel (COMBO) was conducted in Denmark, Russia, and the USA [28••]. In this COMBO study, 13 patients with advanced NSCLC were treated. The primary end point was to describe the side effect profile of the combination treatment and establish the maximum tolerated dose of naptumomab estafenatox in combination with docetaxel. Secondary end points included pharmacokinetics, immunological responses, changes in anti-SAg (anti-SEA/E-120) levels, and effects on tumor disease. Patients were treated with a bolus injection of naptumomab estafenatox in 5 min for four consecutive days followed by 75 mg/m^2^ docetaxel on day 5. Three weeks later this procedure was repeated. Three patients received naptumomab estafenatox at a dosage of 10 μg/kg/day, three patients received it at a dosage of 16.5 μg/kg/day, and seven patients received it at a dosage of 22 μg/kg/day. No synergistic toxicities were observed. The best overall responses were two patients with partial response, five patients with stable disease, and three patients with progressive disease, and three patients were not evaluable. Biopsy specimens were taken from two patients on day 3 of the second cycle. In both patients tumor 5T4 expression and marked T-cell infiltration of the metastases were confirmed. One of these patients had a partial response. The median survival for the patients was 9.3 months. Formation of antibody against SEA/E-120 was low in these patients. Anti-SEA/E-120 levels were compared after the first treatment cycle in the COMBO study with those in the MONO study, and the comparison showed that docetaxel appeared to reduce anti-SAg antibody production when it was given 1 day after the last injection in a treatment cycle with naptumomab estafenatox. Since the avoidance of high-titer neutralizing antibodies would be advantageous for multicycle treatment with naptumomab estafenatox, combinations with docetaxel or other inhibitors of anti-SAg antibody production might be beneficial.

## Naptumomab Estafenatox—Phase 2/3 Studies and Baseline Biomarkers for RCC Patient Selection

In preclinical models, IFN-α had been shown to enhance and sustain TTS-induced CTL activity when given in combination [30]. This observation led to a randomized phase 2/3 trial of naptumomab estafenatox plus IFN-α versus IFN-α conducted in 513 patients with advanced RCC. The patients were recruited between March 2007 and May 2009 and randomized 1:1 in an open-label study to receive naptumomab estafenatox plus IFN-α or IFN-α: 15 μg/kg naptumomab estafenatox was given intravenously in three cycles of four once-daily injections plus IFN-α (9 MU subcutaneously three times weekly) or the same dose and schedule of IFN-α monotherapy alone. The primary end point was overall survival. The secondary end points were progression-free survival, response rate, and safety.

The first set of results was presented at the 2013 American Society of Clinical Oncology annual meeting [31]. Although the primary end point of overall survival (hazard ratio (HR) of 1.08) was not reached, the secondary endpoint of progression‐free survival showed a HR of 0.92. Exploratory endpoints of anti‐SAg (anti‐SEA/E‐120), a biomarker for drug exposure, and IL‐6, a biomarker for immune responsiveness, were assessed as predictive biomarkers. In contrast to the results of earlier studies, significantly increased baseline anti-SAg (anti-SEA/E-120) antibody levels were detected in certain territories. In a subgroup of patients having below median baseline levels of anti-SAg (anti-SEA/E-120) and IL-6, prolonged progression-free survival (HR = 0.62, *p* = 0.016) and overall survival (HR = 0.59, *p* = 0.020) were achieved. Naptumomab estafenatox was well tolerated. Most of the adverse events resulting from treatment with naptumomab estafenatox related to increased levels of cytokines and are expected as part of the mechanism of action. Pyrexia, vomiting, nausea, chills, and back pain were commoner after treatment with naptumomab estafenatox.

A detailed analysis of the results is ongoing and the result will therefore not be discussed further in this review. As previously described, naptumomab estafenatox is a fusion protein containing an engineered hybrid of *Staphylococcus* entertoxins A and E (SEA/E-120). Although SEA/E-120 has been engineered to express a minimum of antigenic epitopes in the wild-type SAgs recognized by patient antibodies at the baseline [12••], certain patients may have elevated levels owing to cross-reactivity to previously encountered wild-type *Staphylococcu*s enterotoxins, e.g., through infections from *Staphylococcus aureus*. The results from the phase 1 studies showed that baseline plasma anti-SAg (anti-SEA/E-120) levels were low in most patients and indicated that the exposure of clinically relevant doses (12 μg/kg or more) of naptumomab estafenatox was independent of the low levels of baseline antibodies. In the phase 2/3 study of RCC patients, the number of patients was vastly expanded and the countries in which they were recruited changed from the phase 1 studies. Increased baseline anti-SAg (anti-SEA/E-120) antibody levels were detected in certain territories, predicting suboptimal exposure [31], which may affect drug activity and antitumor efficacy. Therefore, anti-SAg (anti-SEA/E-120) seems to be a baseline biomarker for exposure important for the selection of patients for treatment with naptumomab estafenatox.

## Conclusion

Naptumomab estafenatox has therapeutic potential in tumors expressing the 5T4 antigen. Clinical development of naptumomab estafenatox is now focused on the identified patient subset of RCC and studies using naptumomab estafenatox add-on treatment with established tyrosine kinase inhibitors in the first or second line. Furthermore, as data accumulate regarding the necessity of using combinations of immunotherapies to reach full effect [32•], naptumomab estafenatox might require the modulation of immune checkpoints with, e.g., ipilimumab or nivolumab, for optimal activity.
